# A Comparative Study of the Perspectives of Long‐Stay Immigrants, Nurses and Cultural Mediators on Intercultural Communication: A Secondary Qualitative Analysis

**DOI:** 10.1002/nop2.70074

**Published:** 2024-11-18

**Authors:** Francesc Ramos‐Roure, Maria Feijoo‐Cid, Josep Maria Manresa‐Dominguez, Jordi Segura‐Bernal, Rosa García‐Sierra, Maria Isabel Fernández‐Cano, Antonia Arreciado Marañón, Eduard Moreno‐Gabriel, Clara Flamarich Gol, Pere Toran‐Monserrat

**Affiliations:** ^1^ Institut Català de la Salut Centre d'Atenció Primària Creu Alta Sabadell Spain; ^2^ Grup de REcerca Multidisciplinar en SAlut i Societat (GREMSAS), (2021SGR1484) IDIAP‐UAB Mataró Spain; ^3^ Departament de Medicina, Facultat de Medicina Universitat Autònoma de Barcelona Bellaterra Spain; ^4^ Facultat de Medicina Universitat Autònoma de Barcelona Bellaterra Spain; ^5^ Unitat de Suport a la Recerca Metropolitana Nord Fundació Institut Universitari per a la Recerca a l'Atenció Primària de Salut Jordi Gol i Gurina (IDIAPJGol) Mataró Spain; ^6^ Faculty of Psychology, Education Sciences and Sport Universitat Ramon Llull Barcelona Spain; ^7^ Grup de Recerca en Comunicacio i Salut (COMSAL) (2017 SGR 922) Barcelona Spain; ^8^ Departament d'Infermeria, Facultat de Medicina Universitat Autònoma de Barcelona Bellaterra Spain; ^9^ Cap Sant Roc Barcelona Spain; ^10^ Departament de Medicina, Facultat de Medicina Universitat de Girona Girona Spain; ^11^ Primary Care Research Group Germans Trias i Pujol Research Institute (IGTP) Badalona Spain

**Keywords:** emigrants and immigrants, intercultural communication, nurse–patient relationship, primary health care, qualitative research

## Abstract

**Aim:**

To compare the perspective of nurses, long‐stay immigrants and cultural mediators on intercultural communication in care encounters.

**Design:**

Qualitative secondary analysis of data obtained in two primary studies.

**Methods:**

Two sets of data from two primary studies on nurses and long‐stay immigrants (including in total two focus groups and 15 in‐depth interviews) were merged. The sample was extended to include a focus group of cultural mediators. An amplified analysis was conducted using Charmaz's approach to grounded theory.

**Results:**

The results are structured under the core category “Agreements and discrepancies in intercultural communication,” split into two subcategories: (1) Communication and the role of culture; (2) (Non) equitable and culturally (in)sensitive care. Immigrant patients and mediators detect barriers associated with generic aspects of communication, while nurses and mediators value culture. Nurses recognise paternalistic attitudes, while long‐stay immigrants sometimes detect biased treatment that mediators do not see. Immigrant patients and mediators value informal conversation as a strategy for cultural learning and building mutual trust, while nurses request regulated training.

**Conclusion:**

The findings show that there are always discrepancies in this relationship. Changes to health care should be based on the participation of all actors. Communication skills training programs should be implemented.

**Implications for the Profession and/or Patient Care:**

The convergences and divergences of nurses, immigrants and mediators expose new ways to approach care. Communication skills training programs should be implemented. Changes to health care should be based on the participation of all actors, including immigrant patients and mediators, and allow them to voice their opinions and make decisions.

**Impact:**

This study addresses intercultural communication from three different perspectives: nurses, long‐stay immigrants and cultural mediators. Nurses, long‐stay immigrants and cultural mediators sometimes show convergence but never completely agree. The research may have an impact on primary‐care nursing by making it more culturally competent.

**Patient or Public Contribution:**

Each participating long‐stay immigrant, nurse and cultural mediator reviewed their own interview. The findings were reviewed by a verifier member of each group (a nurse, patient and mediator).

## 1 Introduction

International migration is a social phenomenon that has displaced more than 272 million people worldwide in search of better living conditions. Arriving in the host country means acquiring new social, communication and cultural skills to adapt to the new society. The process of psychological, cultural and social adaptation of different cultural groups that come into contact is reciprocal, multifactorial and motivates changes in values, beliefs and attitudes (Abraído‐Lanza, Echeverría, and Flórez 2016).

The migration process is a complex phenomenon that encompasses social, economic, cultural and political aspects, among others (Idemudia and Boehnke 2020). Reasons for leaving a country of origin are very diverse. Many people decide to migrate for economic reasons, but they also do so for family reunification or studies. Refugees and displaced people flee political, religious or ethnic conflicts, discrimination based on sexual orientation or identity, adverse climatic and environmental conditions, widespread violence, abuse of human rights, etc. (Idemudia and Boehnke 2020; Fransen and de Haas 2022; Wiegel, Boas, and Warner 2019; Scholten 2022). Furthermore, the demand for qualified labour capital has increased in recent decades (Khalid and Urbański 2021). In the health sector, nurses emigrate to other countries in search of better work conditions and greater growth and professional recognition (Galbany‐Estragués et al. 2024).

Migrants, refugees and displaced persons are vulnerable groups and host countries should guarantee their individual rights and facilitate access to equitable, quality health services. However, these groups frequently encounter barriers to health services (World Health Organization 2019).

## 2 Background

Communication between immigrant patients and health professionals is decisive in care relationships. Barriers to communication make it difficult to access the health system and generate psychological and social problems. Cultural differences and stereotypes cause misunderstandings and relational ship problems that generate frustration and disagreement (Verrept 2019).

Immigrant users perceive ineffective communication in healthcare, discrepancies in health and disease concepts, insufficient information about health, stigmatisation and discrimination. Such circumstances generate insecurity, mistrust, distancing from professionals and the health system. To improve care communication, some immigrant patients use informal interpreters who often omit information, hinder communication, or act on behalf of the patient (Zendedel et al. 2018). To overcome these barriers, professional cultural mediation services in health care were established in Catalonia at the end of the 1990s. Cultural mediators manage the language and facilitate understanding between cultures by creating a safe and conciliatory environment (Marques, Vieira, and Vieira 2019).

The evidence indicates that it is essential to incorporate cultural issues in health care (Liu, Gill, and Li 2021). Communication is inherent to culture and determines the way we relate, transmit, interpret information and express our emotions (Choi, Cook, and Brunton 2019). Intercultural communication—the exchange of information between people with different cultural backgrounds—is considered competent when it is sensitive and framed in the cultural context of the other. In the field of health care, most publications on intercultural communication focus on refugees, recently arrived migrants, or the general immigrant population (Zeidan et al. 2019) and neglect the experiences of long‐stay immigrants—people born abroad who have been settled in the host country for more than 5 years (Malmusi, Jansà, and del Vallado 2007), most of whom are linguistically competent.

Based on the foregoing, intercultural communication is a complex process. Studies have been conducted on the independent perspective of nurses and immigrants but not on that of both actors at once and alongside cultural mediators. Given the complexity of this process and the lack of scientific evidence, this phenomenon needs to be addressed from the perspective of all the actors involved in order to offer a complete picture. Taking all this into account, we ask ourselves: What is intercultural communication like in primary care in the nurse–immigrant patient relationship? The purpose was to contrast the points of view of the actors involved to explore whether there are points of agreement and divergence.

## 3 Aim

The aim of this study is to compare the perspectives of nurses, long‐stay immigrants and cultural mediators on intercultural communication in care encounters.

## 4 Methods

### 4.1 Design

This is a qualitative secondary analysis of data obtained in two primary studies (Ramos‐Roure et al. 2021; Torán‐Monserrat et al. 2013) and data newly collected for the new study's purpose. A qualitative secondary analysis is defined as the use of an existing qualitative data set to answer a different research question than that posed by the original or primary study. We opted for a secondary analysis, basically due to the difficulty in accessing these immigrants and so as not to overwhelm the vulnerable populations that participated. We aimed to maximise the value of participants' contributions by reusing existing data (Ruggiano and Perry 2017; Chatfield 2020). In this secondary analysis, an amplified analysis was performed since data sets from two primary studies were combined with new primary data—thus amplifying the sample for comparison and analysis—to respond to our research purpose (Heaton 2008).

### 4.2 The Primary Studies

The data sets analysed belong to two primary studies conducted at the primary care centres of the Maresme region (Catalonia, Spain) by the researchers of the current study.

Firstly, data from phase two of the project PROMISE were used, corresponding to one focus group and 10 semi‐structured interviews with primary care nurses. In 2013, a study protocol named PROMISE began. It aimed to provide better knowledge of psychological distress in the immigrant population and to explore the efficacy of health care providers with respect to both communication and the therapeutic management of these situations. The aim of this first primary study was to explore the professional skills necessary to offer culturally competent care based on the concept of critical incident (Torán‐Monserrat et al. 2013). The script for these interviews explored three scenarios of the encounter/communication with immigrant patients: everyday situations in the nursing consultation, complex situations (difficult to resolve) and problematic situations (with major or irresolvable obstacles). Flanagan's (1954) critical incident technique was used in the interviews with the nurses. Flanagan defines an incident as critical when the context in which it occurs and its consequences are important to the interviewee. The participating nurses were of Spanish nationality, had Spanish/Catalan cultural background and spoke Spanish and/or Catalan (both languages are co‐official in Catalonia), had an average of 18.5 years of experience in primary care and cared for an average of 33 immigrant patients per week (see Table 1). Two of the researchers of the current study were involved in the data collection for this primary project. As a result of an initial narrative analysis of the data, the following themes were identified: difficulties in nurse–patient communication; barriers associated with language and culture; attitude and disposition of the nurse to care for the long‐stay immigrant; and skills and knowledge necessary for such care. These results were not published partly due to the passing of one of the researchers.

**TABLE 1:** Description of the participants.

Participants	Interviews	Focus groups
Nurses	10	8
Age (average)	50.5	45.7
Females	9	8
Males	1	0
Years in primary care (average)	20.4	16.8
Immigrants cared for per week (average)	26	42
Training in multiculturalism and/or migration	1	4
Long‐stay immigrants	5	8
Age (average)	48	41.8
Females	3	4
Males	2	4
Years in Spain (average)	20	13.2
Cultural mediators	0	4
Age (average)	—	50
Females	—	3
Males	—	1
Years in Spain (average)	—	23

As for the second primary study, partly published in 2021 and aimed at exploring the experiences and perspectives of long‐stay immigrants on intercultural communication in encounters with primary care nurses, this study consisted of one focus group and five interviews is used in the present study (Ramos‐Roure et al. 2021). Purposive sampling was used to recruit long‐stay immigrants following these criteria: over 25 years of age, more than 5 years of residence in Spain, fluency in Catalan or Spanish, originating from the Andean, North African, sub‐Saharan and South Asian regions and China. As they were long‐stay immigrants and had been in the country for more than 5 years, all participants spoke in Spanish and cultural mediators were not needed to translate. The group of immigrants had a mean age of 44 years and 17 years of residence in the country (Table 1). Following Charmaz's constructionist approach to grounded theory, the analysis of this second primary study offers a detailed description of how long‐stay immigrants experience and perceive intercultural communication in encounters with primary care nurses.

Long‐stay immigrants from the second primary study reported the desire for closer, more personalised and humanised communication but did not consider culture important to the communication process. The nurses brought up critical incidents related to language and culture. Consequently, it was deemed necessary to re‐examine and compare the data from nurses and long‐stay immigrants to explore similarities and differences in intercultural encounters in greater depth, as well as collecting further data from cultural mediators. In our setting, cultural mediators are trained in dialogue skills, conflict management, the development of egalitarian relationships and the promotion of social change. We chose to include their perspective because they offer an external, complementary and perhaps more objective view of the nurse–patient relationship.

### 4.3 Population and Sample

As the focus of the study was to compare the perspectives of the actors involved in intercultural communication in health care, we purposefully selected all the transcriptions of interviews with nurses (*n* = 18) and immigrants (*n* = 13) (see Table 1) from the primary studies. The cultural mediators had to be from one of the regions of the long‐stay immigrants, work as cultural mediators in health care in Mataró (Maresme), have training in cultural mediation. The municipality of Mataró invited the cultural mediators to participate (see Table 1).

None of the researchers knew the study participants in advance in either the primary studies or the current study. The cultural mediators received a travel card to cover travel expenses.

### 4.4 Data Sources and Collection

**FIGURE 1:**
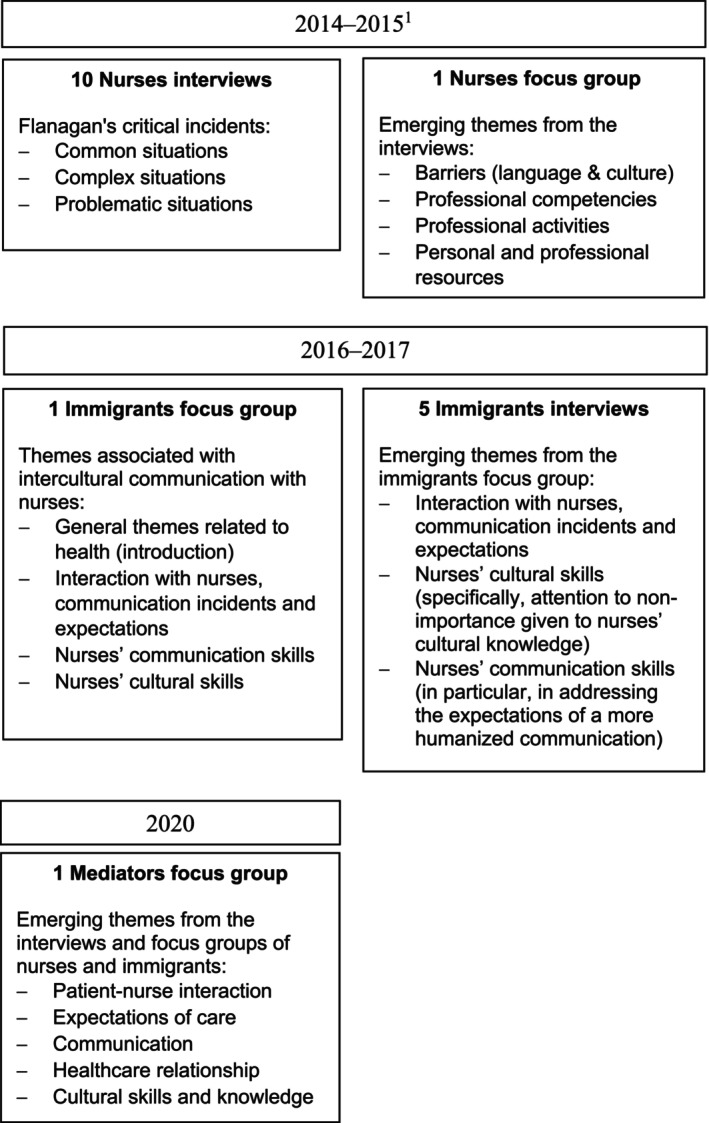
Data collection scheme. ^1^These data on the professional skills needed to provide culturally competent care belong to the 2013 project. No publication associated.

The data from the primary studies were collected as follows: the nurses data set were collected in 2014–2015; the long‐stay immigrant data set was collected in 2016–2017. The interviews from the primary studies lasted 30 to 85 min and the focus groups lasted 90–105 min. The data collection sequence can be seen in Figure 1. The same researcher was present at the focus groups of nurses, long‐stay immigrants and cultural mediators in the role of group conductor or observer. This researcher also conducted the interviews with the long‐stay immigrants.

The primary data from cultural mediators was collected in 2020. We planned one focus group with six cultural mediators to understand the issues on intercultural communication on which nurses and patients differed and/or which had not been sufficiently explained. Only four attended in the end. The script for the focus group was prepared based on the re‐analysis of the transcribed interviews with nurses and long‐stay immigrants. Similarities and differences were identified to be discussed with the cultural mediators. The script included questions such as “Some nurses feel that sometimes they and the patients do not understand each other (even when they speak the same language), can you explain why it happens?”; “Some patients identify prejudices and inappropriate attitudes among some professionals (e.g., ignoring the patient, a lack of respect, contempt, etc.). Tell us about your experience with this”, “Many long‐stay immigrants think that the inclusion of cultural aspects in medical and nursing care is not important, what is your opinion?”. All interviews were audio‐recorded. Field notes were taken by the observer during the focus group.

### 4.5 Data Analysis

Reanalyzing the data meant integrating three data sets under a single view defined by the new research aim. The steps laid out by Alvesson y Kärreman were followed to do so (Alvesson and Kärreman 2007). Since part of the research team was already familiar with the topic of interest—professional competencies in care for immigrant patients in project PROMISE from the perspective of nurses and intercultural communication from the perspective of immigrant patients—, the primary data were studied and a process of reflection was initiated. Afterward, the research aim was formulated to compare the data sets from both studies. The purpose was to compare the perspectives of the parties involved in intercultural communication in care encounters. During the data integration process, data was compared and analysed to detect similarities and discrepancies. The data were analysed once again, and all the information was reassessed and reinterpreted to make sense of the points of conflict that arose.

A test of the quality of the primary data was carried out (Sherif 2018), as can be observed in Table 2, in which the following was assessed: (1) the appropriateness and pertinence of the data set to the current study (connection between the data sets and the aim of the secondary study); (2) the overall quality of the data (availability, accessibility, depth, breadth, completeness and accuracy of the data set, methodology and instruments); (3) reliability (complete information on the primary studies, researchers, data collection methods); and (4) data set timelines (timeline of the research, data collection and analysis, temporal validity).

**TABLE 2:** Evaluation criteria in the secondary analysis of qualitative data.^a^

*Instructions*. Circle^b^ each criterion of preexisting qualitative data using the following rubric. A cumulative sum of circled^b^ criteria is used to suggest the extent of data quality, sufficiency and fit for secondary analysis.		
Assessment Criteria		
Fit and relevance of dataset to present research		
Fully met	Partially met	Not met
Preexisting data are centered around only topic of interest. Topic of interest is logically linked to dataset. Secondary research questions are built upon aims and objectives of primary study. Participants of original study describe/report on issue of interest. Participants of original study report/describe various aspects of topic of interest. There is strong evidence that participants of original study experienced topic of interest. Original research background is relevant to topic of interest. Secondary research questions are written broadly to limit the influence of personal biases on data reading.	Preexisting data are centered around the topic of interest along with other topics. Topic of interest is somewhat logically linked to dataset. Dataset contains information to partially answer secondary research questions. Participants of original study briefly describe/report on topic of interest There is some evidence that participants of original study experienced topic of interest. Original research background is somewhat relevant to topic of interest. Secondary research questions are written somewhat broadly.	Preexisting data have little or no evidence of topic of interest. Topic of interest is not linked to dataset. Dataset has little or no information to fully answer secondary research questions. There is very little or no evidence of participants of original study experiencing topic of interest. Original research background is not at all relevant to topic of interest. Secondary research questions reflect personal biases or predisposition of secondary research findings.

General quality of dataset		
Fully met	Partially met	Not met
Preexisting data are rich and descriptive of topic of interest. Data include participants' insights, experiences, and reactions to topic of interest. Data are collected via numerous data collection instruments. Secondary researcher, if not the author of primary study, has full access to dataset and its accompanying documentation. Dataset documentation (tapes, transcripts, protocols, notes) is sufficient to fully answer secondary research questions. Dataset documentation (tapes, transcripts, protocols, notes) is present in sufficient quantity that allows for data saturation. Dataset documentation reflects the type of sample, its size, demographics and, if possible, geographic descriptors, recruitment and consent procedures. Transcription of interview/focus group data is accurate and has no or minimal typographical errors, incomplete sentences, and/or missing words. Interview/focus group transcriptions are accompanied by transcription protocols that include instructions for transcribers and decisions addressing inaudible text segments, overlapping speech, unfamiliar terminology, and language‐specific nuances. Dataset includes many instances of information restating, summarizing, and/or paraphrasing of participants' insights to assure collected data are correctly recorded and understood. Access to participant contact details is available to only the author of primary research. When secondary analysis research is conducted by the author of primary study, consent form allows for reconnecting with study participants to clarify characteristics of original research and/or complete missing information.	Preexisting data are somewhat descriptive of topic of interest. Data include participants' incomplete/underdeveloped insights and limited experiences of topic of interest. Dataset consists of two or three types of data. Secondary researcher, if not the author of primary study, has partial access to dataset and its accompanying documentation. Dataset documentation (tapes, transcripts, protocols, notes) is sufficient to only partially answer secondary research questions. Dataset documentation (tapes, transcripts, protocols, notes) is present in somewhat sufficient quantity that allows for partial data saturation. Dataset documentation reflects the type of sample, its size and demographics. Transcription of interview/focus group data has some typographical errors, incomplete sentences, and missing words making generation of meaning challenging. Dataset includes some transcription protocols with some instructions for transcribers to address inaudible text segments, overlapping speech, unfamiliar terminology, and language‐ specific nuances. Dataset includes some instances of information restating, summarizing, and/or paraphrasing of participants' insights to assure collected data are correctly recorded and understood.	Preexisting data have minimal or no details about topic of interest. Participants' insights and/or experiences are irrelevant to topic of interest. Dataset consists of one type of data (interview, focus group, observational, or documents). Secondary researcher, if not the author of primary study, has limited access to collected data. There is little or no access to the documentation, accompanying the original study (research background description, sample design, data collection protocols, etc.). Dataset documentation (tapes, transcripts, protocols, notes) is presented in limited quantity and allows for little or no data saturation. There is little evidence of the type of sample, its size or demographics. Transcription of interview/focus group data has many typographical errors, incomplete sentences, and/or missing words making generation of meaning impossible. Dataset includes few or no transcription protocols with little or no instruction for transcribers to address inaudible text segments, overlapping speech, unfamiliar terminology, and language‐specific nuances. Dataset has a couple or no instances of information restating, summarizing, and/or paraphrasing of participants' insights to assure collected data are correctly recorded and understood.

Trustworthiness of dataset		
Fully met	Partially met	Not met
Dataset includes detailed description of primary research questions, study aims and objectives. Dataset includes detailed description of primary research timeframe, its settings, and data collection settings. Dataset includes credentials and institutional affiliations of team members who conducted primary research.	Dataset includes some description of primary research questions, study aims or objectives. Dataset includes some description of primary research timeframe, its settings, or data collection settings. Dataset includes incomplete credentials or institutional affiliations of team members who conducted primary research.	Dataset includes little or no description of primary research questions, study aims or objectives. Dataset includes little or no description of primary research timeframe, its settings, or data collection settings. There is no information about credentials or institutional affiliations of team members who conducted primary research.

Timelines of dataset		
Fully met	Partially met	Not met
Secondary researcher, if not the author of primary study, has full access to timeline of research initiation, data collection, and analysis. Data points and protocols of data collection are time‐stamped. Data are current and/or relevant to present day topic of interest.	Secondary researcher, if not the author of primary study, has some access to timeline of research initiation, data collection, or data analysis. Either data points or protocols of data collection are time‐stamped. Data are somewhat current and/or relevant to present day topic of interest.	Secondary researcher, if not the author of primary study, has limited or no access to timeline of research initiation, data collection, or analysis. Data points and protocols of data collection are missing time stamps. Data are outdated comparing to present day topic of interest.

^a^ Sherif, V. (2018). Evaluating Preexisting Qualitative Research Data for Secondary Analysis. *Qualitative Social Research*, 19(2), 1–17.

^b^ In the original instructions, author recommend to circle but to better see as a table we made a 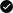.

Two researchers with an expertise in qualitative methods performed the data analysis together using Charmaz's approach to grounded theory analysis. The recordings were transcribed verbatim and all the data were reviewed prior to coding. Using the qualitative data analysis software ATLAS.ti, open coding was started, the data was coded line by line, highlighting important concepts and phrases to later continue with focused coding selecting the most significant and/or frequent open codes that were grouped into more general codes, thus establishing the analytical categories. Finally, selective coding highlighted the core category (“Agreements and discrepancies in intercultural communication”) and its relationships with other categories. The constant comparative method was used at each level of analysis. The constant comparison between the categories allowed us to generate a core category, identified through an iterative process in several meetings between the researchers. Coding and interpretation were conducted jointly by both researchers (M F‐C & F F‐R) in an iterative process with the aim of taking an in‐depth look at the topics of interest that were emerging. Discrepancies were discussed by the research members until reaching a consensus. Two researchers independently reviewed the coding process and results (see Table 3).

**TABLE 3:** Overview of the coding process.

Core category	Agreements and discrepancies in intercultural communication		
Categories	Communication and the role of culture		(Non) Equitable and culturally (in)sensitive care
Subcategories	(Non) Importance of culture	Communication skills and commitment of nurses	
Codes	Language Different cultural codes Nurse's disposition Acculturation Multicultural Baggage Concepts: health and illness	Misunderstandings Empathy and patience Sensitive care Active listening Informing Professionally adapting Commitment and professional involvement Time spent with the patient Cultural openness Cultural curiosity Education and empowerment Consensus	Respect Paternalism Care expectations Not trusting the professional Avoiding confrontations Traditional therapies Informal conversation Building trust Inappropriate attitudes in the nurse Biased attitudes Incidents

### 4.6 Ethical Considerations

The PROMISE study—initially involving nurses only—obtained approval from the Clinical Research Ethics Committee of the Jordi Gol Primary Care Research Institute. As further data collection, both from long‐stay immigrants and cultural mediators, was deemed necessary for addressing this study's original research questions, subsequent extensions were asked and approved by the same Ethical Committee. Participation was voluntary and informed consent documents were signed by participants in all primary studies.

Regarding whether informed consent can be assumed for the secondary analysis presented here, we believe that it can draw on the following arguments. Firstly, as several academics have pointed out, it is not necessary and even potentially unethical to seek further consent (Grinyer 2009) in cases such as this, where any new questions do not shift the initial focus of the research (i.e., data will still be used to produce knowledge on intercultural communication and not for other purposes) (Long‐Sutehall, Sque, and Addington‐Hall 2011). Furthermore, the research participants generously gave their time to supply information—and the time required for qualitative research can be considerable—and therefore expect use to be made of the results (Corti, Day, and Backhouse 2000).

### 4.7 Rigour

To strengthen confirmability, two researchers who were not involved in data collection or analysis reviewed the analysis and coding process and discussed the results from an external perspective. One of these researchers is an expert in migration issues and a researcher in the primary study PROMISE and the other is an expert in qualitative methodology. Reliability was reinforced by the accuracy and description of the primary studies and the design of the secondary analysis. To avoid interpretations that were influenced by the cultural background of the researchers, the results were reviewed by a checking member from each group (a nurse, a patient and a mediator). A nurse external to the study with expertise in primary care and migration also reviewed the findings. The analysis and quotations from the participants illustrating the main themes bolstered the consistency of the findings.

The reliability of reanalyzed preexisting data may be called into question. However, the exhaustiveness of the description of the primary studies increases the quality and adequacy of the data and offers a detailed understanding of the peculiarities of the primary research and its theoretical/practical contribution to knowledge (Sherif 2018). The research question explored through qualitative secondary analysis “fits well” with those of the primary studies, so increasing the reliability of the results. The COREQ checklist (see Appendix S1) was used in the study (Tong, Sainsbury, and Craig 2007).

## 5 Findings

Through data analysis, we identified two main categories. (1) communication and the role of culture; (2) (non) equitable and culturally (in)sensitive care. These categories interact with each other and come together in the core category of this study, “Agreements and discrepancies in intercultural communication,” which shows the differences and similarities in communication expectations in encounters between long‐stay immigrants and nurses and what was observed by the mediators.

### 5.1 Agreements and Discrepancies in Intercultural Communication

**FIGURE 2:**
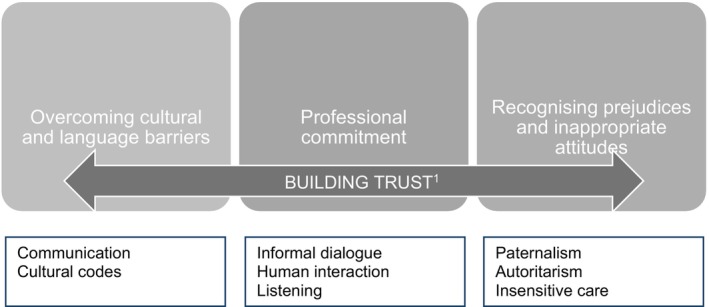
Agreements and discrepancies in intercultural communication. ^1^Our participants agree in that building trust is key in intercultural communication and can be achieved by way of informal dialogues that show nurses' commitment and sensitive care. On the other hand, each group highlighted different barriers or threats to an effective intercultural communication. We have attempted to represent this dynamic and contentious nature in this figure.

This core category describes discrepancies or agreements between nurses, long‐stay immigrants and cultural mediators regarding fundamental aspects of intercultural communication and the healthcare relationship. This core category suggests that intercultural communication is far from a clear‐cut process or reality, but a contested and controversial tension, always being negotiated and emerging from the interacting views of all the stakeholders involved (see Figure 2).

#### 5.1.1 Communication and the Role of Culture

##### 5.1.1.1 (Non) Importance of Culture

Nurses, long‐stay immigrants and cultural mediators agree that language is the main barrier to care in the years following arrival. Linguistic competence is acquired progressively, although language problems persist in some people. However, nurses and cultural mediators find that barriers to communication go beyond language and that both parties may fail to understand each other even when patients' mother tongue is Spanish.

Despite speaking the same language, there are also communication problems with South Americans because we have very different concepts [about illness, chronicity, care expectations, etc.] (Nurse‐GF4)

Cultural mediators consider culture to be the main barrier to communication because it is complex, multifaceted and persistent over time. In fact, cultural mediators do not speak of culture but of cultural codes. They posit that nurses and immigrants have different codes that can make communication difficult. For them, cultural codes are the multiple meanings that give sense to everyday life and include one's view of life and death, ways of relating to others, social and family values, etc. An intercultural relationship involves understanding, learning and managing new cultural codes to deal with everyday interpersonal situations and achieve effective communication and a satisfactory care relationship.

[As a cultural mediator] You have to anticipate what might happen in the consultation and explain it to patients to avoid misunderstandings: ‘When you go to the doctor and explain your problem to them, don't misunderstand if they react this way or that’. (Mediator‐GF1)

On the other hand, most long‐stay immigrants do not consider cultural differences to be of importance but rather the inadequate predisposition of nurses towards dialogue and interacting. A Moroccan patient residing in Spain for 30 years said:

It doesn't matter whether you are Chinese, Moroccan, Senegalese, or Gambian; it has nothing to do with culture. If you are sick, you want to be treated well, like a person. (Patient‐FG1)

##### 5.1.1.2 The Communication Skills and Commitment of Nurses

Once the language barrier is overcome, patients and nurses confirm that they express themselves clearly even though they do not always understand each other, blaming the other for the lack of understanding. Nurses are recognised for their empathy and professionalism—essential skills for caring for people. However, immigrant patients perceive a lack of empathy and mechanised care in nurses, which they attribute to care pressure and stress. Cultural mediators support the testimony of immigrant patients, although they attribute it to the management of different cultural communication codes, technical language, a lack of compassion on the part of some professionals and insufficient communication time for the patient. On the whole, these factors lead to misunderstandings, diminish trust and negatively condition successive visits.

Nurses lack empathy and patience. They are not workers hammering nails; they work with human beings. And human beings are sensitive people, not objects. (Patient‐E2)

We need to put ourselves in the place of others, a little empathy. Doctors have a full list [of patients to visit] and care is cold and insensitive. (Mediator‐GF1)

Patients and mediators perceive deficiencies in nurses' listening, exchange of information and communication. Immigrant patients expect to receive clear, useful and simple information. Many of them consider it an unmet need. Cultural mediators add that clarity in explanations, avoiding technicalities, showing patience and encouraging the patient to express emotions facilitate communication, help prevent misunderstandings and improve the mood. The nurses also observe that immigrant patients need to exchange information and they take advantage of this circumstance to improve communication and strengthen the relationship.

Professionals have a long list [of patients to visit] and they don't have time to listen to everyone: the patient, an interpreter and a mediator. The patient needs to be heard, to be understood. (Mediator‐GF3)

In a routine visit, you don't have to ask anything. People open up a lot and explain things about family and work. (Nurse‐FG4)

Immigrants believe that nurses show only limited involvement in solving their health needs, except in paediatrics and psychiatry. Immigrants believe that professional involvement means showing motivation, concern and commitment; it means that the patients perceive an interest in their personal case, culture and values and feel listened to and included in their care. In contrast, nurses express their full commitment to immigrant patients in the form of support and empowerment to achieve optimal levels of health. In complex situations, such as ablation, some nurses consider committing themselves to the point of ethical limits. One nurse described how her emotional and professional commitment prevented the ablation of two of her patients:

They wanted to cut the girls in Africa (…). I wanted to report it, but I would lose the mother's trust (…). I contacted an association of African women, they spoke with the father (…) and, supported with a letter informing about the prohibition of performing female circumcision, the girls returned safe and sound. (Nurse‐E12)

#### 5.1.2 (Non) Equitable and Culturally (In)sensitive Care

Respect is the most valued quality among immigrant patients. Despite wanting an equal, friendly and humanised relationship, immigrant patients perceive biased and prejudiced attitudes among some nurses (unequal treatment, distance, prejudice, etc.). In contrast, cultural mediators rarely observe stigmatisation, prejudice, or imposing attitudes among professionals. However, they perceive mistrust towards health professionals in some patients, which, added to cultural differences and care expectations, leads them to interpret the treatment they receive as biased.

Sometimes they treat you very rudely. The first time, you try to excuse her (…) The third time, you think it's because I'm South and she sees me badly. (Patient‐E2)

‘Because I'm black, I can take more pain and that's why they don't treat me.’ You try to explain to them that everything has its own process, but they continue to have that perception. (Mediator‐GF4)

Immigrant patients first justify prejudice or unequal treatment from professionals on the basis of work stress or personal problems. However, when it happens repeatedly, they link it to racism. On other occasions, immigrant patients feel intimidated by the authoritarian attitudes of some professionals, although they rarely show disapproval in public. Cultural mediators believe that immigrant patients avoid conflict to protect themselves from buried emotional trauma or out of fear of having worse experiences with a new professional. A change of professionals would mean explaining their medical history and the vicissitudes of their migration process once more, which they may not always want to relive, even more so when trust has not yet been established with the professional.

Some women have been raped during migration and they don't want to be reminded of it again with a new person. (Mediator‐GF3)

The nurses ensure that they treat all patients equally and respectfully, without ethnic or cultural distinction. Even so, they acknowledge taking paternalistic and authoritarian attitudes when they do not have time for health education, to seek consensus, or when they interpret that a patient is not responsible for managing their own health.

I threaten them that it's time for a vaccine and then they come every year. I trick them a little and that's how I check the child's health. (Nurse FG4)

Regarding nurses' dominant position, mediators observe that, at times, patients are treated arrogantly; they tend to avoid confrontation or else, they quarrel over minor issues. In fact, when faced with conflict, long‐stay immigrants feel vulnerable and inferior. They do not demand their rights as healthcare users, but rather avoid conflict.

They [patients] simply tell you: “I don't want any more problems.” Because if I complain, then there will be problems, they will treat me badly, it will affect my papers and that will appear in all the databases. Even the police will know that I have been complaining here. (Mediator‐GF2)

Only Muslim patients report that nurses adapt care to their religious beliefs—during Ramadan. The mediators believe that nurses are not flexible when they act paternalistically, offer care that is not culturally adapted and ignore traditional therapies. Cultural mediators point out that standardised care is a form of social pressure to renounce cultural identity. Very few professionals agree with patients on the use of alternative therapies or accept superstitions or amulets.

When you arrive in a new culture, you are lost, you don't know what to choose, one way or another. [host country] asks you for total integration, but that does not exist. You cannot forget what is yours, as if what you carry in your backpack is of no use. And even more so if you come from the third world, because they think you don't understand anything and you don't know anything. (Mediator‐GF2)

Informal dialogue emerges as a tool to break the ice, to become aware of the other person and to get to know their way of seeing reality. Mediators and patients agree that informal conversation is a key element to building a relaxed, horizontal and trusting relationship. They agree that building trust is important in intercultural communication and can be achieved by way of informal dialogues that show nurses' sensitive care. Immigrant patients believe that informal communication is less dictatorial, facilitates closeness and improves communication and relationships. For them, active listening and conversation are informal tools for learning and understanding the world of immigrant patients. This is due to the interest shown in the other person, the exchange of cultural and personal information—regarding gastronomic, cultural and religious habits—, and each party learning from the other. Immigrants aspire to more interaction in this regard.

You treat me well if you simply ask me: “It's raining, right? It's so cold!” This is how you move from a cold conversation, because I don't know you and you don't know me. But the fact of asking makes me understand that I exist and that you see me. (Patient GF1)

(…) you have to talk to people and get to know them (…). We also learn things when we talk to you. (Patient‐E1)

Cultural mediators equate informal conversation with icebreaking rituals and ways to make the other feel visible accepted. This is how one mediator expresses it:

If during first meet, [the health worker] we have a simple conversation, then the ice is broken, a connection is created, the person feels that they exist and they feel accepted. (Mediator‐GF1)

## 6 Discussion

This is one of the scarce pieces of evidence that explores the perspective of all the actors involved in intercultural encounters at once. The findings show that immigrant patients, nurses and mediators have very different perceptions of intercultural communication. Immigrant patients and mediators detect barriers associated with generic aspects of communication (lack of active listening, inattention, emotional aspects, etc.) and value informal dialogue as a strategy for learning and building trust while nurses and mediators value culture in the communication process.

### 6.1 Communication Skills of Nurses and the Role of Culture

The results of this study show that most long‐stay immigrants focus their attention on communication and the care relationship with nurses and relativize cultural aspects. Both facts have been previously described in the literature: immigrant patients who have been in the country for a longer time are less satisfied with the level of listening (Han and Lee 2016). Previous evidence of both long‐stay immigrants and the immigrant population in general points to the relative weight of culture in the care relationship. First‐ and second‐generation immigrants prefer a respectful, humanised and functional relationship (Tavallali, Jirwe, and Kabir 2017). Many authors have already underlined the importance of culture in the care relationship. Health professionals express the need to acquire knowledge about cultural diversity to overcome differences in the representations of health and disease, although they also express the difficulty of providing biomedical care (Shepherd et al. 2019). Paradoxically, the nurses in this study report that they adapt care to the patient's culture, while the mediators point out that traditional healing elements are not integrated.

### 6.2 ON (Non) Equitable Care and Informal Conversations

The results reveal that immigrant patients perceive a power imbalance in the relationship and paternalistic treatment by nurses. The international literature confirms the existence of paternalistic, imposing, and prejudiced attitudes of health professionals and the biomedical health system (Kanengoni, Andajani‐Sutjahjo, and Holroyd 2020). More than 60% of immigrants residing in Barcelona perceive discrimination in health care (Velasco, Vinasco, and Trilla 2016), while some professionals see immigrants as a threat to public health (Madeira et al. 2018). There is literature that negatively associates prejudiced and stereotyped attitudes of professionals with professional communication styles, misunderstandings, non‐verbal communication, time dedicated to patients, treatment recommendations and trust (Schnierle, Christian‐Brathwaite, and Louisias 2019). In contrast, cultural mediators rarely observed biased attitudes among nurses, although they did observe that some immigrant patients had a prejudiced image of professionals and the health system. Although it could also be because cultural mediators have been in the host country for many years, are part of the health system and defend a fair outcome to the conflict from an evaluative approach, knowing that resolving the conflict will be difficult (Ng 2016). Sometimes the power imbalance can be incorporated by cultural mediators as a tool to improve and accelerate the negotiation (Ng 2016). The discrimination perceived by immigrant patients is related to the degree of acculturation: immigrants who adopt adaptive strategies—greater language competence and greater socialisation in the host group—perceive greater discrimination. However, in immigrants with an unfavourable socioeconomic context—social isolation, administrative status, job insecurity, etc.—segregation strategies and mistrust of the health system increase the risk of social vulnerability, discrimination and distancing from the healthcare system (Oakley, López‐Cevallos, and Harvey 2019). Although the process of social, cultural and psychological adaptation should be mutual, the host society is unable to accommodate the new culture and limits it to the position of the “other,” which is interpreted as an act of discrimination or racism (Choi, Cook, and Brunton 2019).

Some nurses in the study admitted to having knowingly dispensed paternalistic and authoritarian treatment. Paternalistic communication makes understanding difficult and limits the patient's autonomy in decision‐making. Various explanations may make sense of conscious paternalistic treatment by nurses: (a) demonstrations of paternalism are accepted in the Mediterranean culture (Gironés 2015); (b) the perception of vulnerability generates protector attitudes that depart from empowerment and shared decisions (Gironés 2015); (c) the patient's inclination towards paternalistic models that delegate decision‐making to the health professional (Thompson and Whiffen 2018).

For patients and cultural mediators in the study, the wish to establish an informal conversation during the professional‐patient encounter is a particularly relevant issue because it is a key element to building a relaxed, horizontal and trusting relationship. Immigrant patients believe that informal communication is less dictatorial, facilitates closeness and improves communication and relationships. Some authors point out that informal conversation has cultural and social connotations, it helps to relax the relationship, yield a holistic understanding of the patient and their environment, and promote social interaction and the exchange of information (Chen et al. 2013). In contrast, formal communication is cold, structured and neglects the very human aspects of the encounter (Chen et al. 2013). Renshaw (2004) argues that the Dialogue as Conversation aims to establish a mutual understanding, intersubjectivity and consensus. Freire, pointed out that dialogism is inherent to human nature and a requirement for democracy (Aubert and Soler 2007). We use dialogue to build meanings, acquire knowledge and skills, and take action, but also to live together on the basis of equal rights—it is necessary to talk and reach agreements. Long‐stay immigrants and mediators further argue that this type of informal conversation would allow each party to learn from the other; this could be framed in what literature calls dialogic learning. Dialogic learning is the fruit of interactions in an egalitarian dialogue where, based on consensus, new knowledge is created and acquired (Racionero and Valls 2007). This learning does not occur in power relationships but in dialogical ones, where participants contribute the knowledge, they got from their experience and skills, in an equitable way, with the intention of mutual understanding and build on shared agreements to collectively create learnings.

### 6.3 Strengths and Limitations

As the study involved a secondary analysis of data from previous studies, the analysis was limited to the data originally collected. These data were collected in specific temporal and socio‐political contexts that may have conditioned both the data as well as the interpretation thereof—a reality that may be clear at the time of collecting the data but go unnoticed years later. However, the continuity of the researchers in all the studies lessens this limitation. Being unable to return to the field certainly limits the addressing of new questions, but this is an issue with the majority of research, whether primary, secondary, qualitative or quantitative (Hammersley 2010). Nevertheless, re‐analysis can allow for unexpected facts and data to be registered.

The previous data collection responded to specific objectives different from those of the current study, which may limit the depth of study of the new aim or the extent to which a thematic conclusion could be identified. Nevertheless, the research question for this study fits well with the purpose of the original studies: they all deal with intercultural communication between professionals and immigrants. Moreover, the collection of new data from the group of cultural mediators (from the perspective of observers of intercultural encounters trained in mediation) acted as a control mechanism to determine if the results did not correspond to reality or, on the contrary, verified the results.

We didn't take into account “the years of experience as cultural mediators” as a selection criterion due to both the difficulty in recruiting these participants and the shortage of mediators. This limitation can influence the way cultural mediators view and interpret communication, as well as the influence of culture on communication during the research work.

The various stakeholder groups differed greatly in size and there was no diversity of country of origin of nurses (they were all Spanish), which may limit the transferability of the results. However, the study presents data from (1) immigrant patients, a population group considered vulnerable, (2) cultural mediators, who have been vanishing from the health system and for which there is little evidence and (3) there were few foreign nurses in 2015 because their degrees were not recognised in Spain, meaning they had to do their qualifications again. Moreover, there are no studies that include all three groups and the results of this study aim to open the door to new research questions.

Not having the informed consent of the participants of the primary studies to carry out the secondary analysis could be a limitation. However, as several authors highlight (Grinyer 2009; Ruggiano and Perry 2017; Chatfield 2020), asking participants to give their consent repeatedly raises practical problems—and sometimes ethical problems when dealing with vulnerable populations. The aim is to maximise the value of participants' contributions by reusing existing data.

## 7 Conclusions

This study provides a critical and plural view of intercultural communication, including the discourse—convergences and divergences—of the actors involved: nurses, long‐stay immigrants and cultural mediators. This multifaceted convergent and divergent perspective highlights new ways to improve the provision of health care to the immigrant population and transform it into culturally sensitive, equitable and respectful care. The implementation of communication skill programs, dialogical learning and the strengthening of links between immigrant communities and the health system, as well as the promotion of the participation of communities and cultural mediators in health‐related processes, is recommended. A process of individual, professional, institutional and community self‐reflection must be started to rebalance the relationship, encourage the integration of strategies and promote equity in the provision of health care to immigrant patients.

## Author Contributions

**Francesc Ramos‐Roure:** methodology, formal analysis, investigation, data curation, writing – original draft, writing – review and editing, visualisation. **Maria Feijoo‐Cid:** conceptualization, methodology, formal analysis, writing – original draft, writing – review and editing, supervision, project administration. **Josep Maria Manresa‐Dominguez:** writing – review and editing. **Jordi Segura‐Bernal:** conceptualization, investigation, data curation. **Rosa García‐Sierra:** validation, writing – review and editing. **Maria Isabel Fernández‐Cano:** writing – review and editing. **Antonia Arreciado Marañón:** writing – review and editing. **Eduard Moreno‐Gabriel:** writing – review and editing. **Clara Flamarich Gol:** writing – review and editing. **Pere Toran‐Monserrat:** conceptualization, methodology, validation, resources, visualisation, project administration, funding acquisition.

## Conflicts of Interest

The authors declare no conflicts of interest.

## Supporting information


Appendix S1

## Data Availability

The data that support the findings of this study are available on request from the corresponding author. The data are not publicly available due to privacy or ethical restrictions.
